# Une tumeur exophytique du cuir chevelu

**DOI:** 10.11604/pamj.2017.28.45.13535

**Published:** 2017-09-18

**Authors:** Ilhame Naciri, Baderddine Hassam

**Affiliations:** 1 Service de Dermatologie et Vénérologie, Centre Hospitalier Universitaire IBN SINA, Faculté de Médecine et de Pharmacie, Université Mohammed V, Rabat, Maroc

**Keywords:** Tumeur trichilemmale proliferante, maligne, cuir chevelu, Proliferating trichilemmal tumor, malignant, scalp

## Abstract

Proliferating trichilemmal tumor (PTT), still referred to as proliferative trichilemmal cyst (TC), is an uncommon malignant adnexal tumor originating from the cells of the outer root sheath of the hair follicle or, more often, from a trichilemmal cyst, following multiple trauma and/or recurrent inflammation. We report the case of a 64-year old female patient, with no particular previous history, presenting with tumor of the scalp gradually evolving over 18 months. Clinical examination showed a painless, firm, adhering ulcero-budding tumor mass measuring 12 cm along its longer axis at the level of the vertex. The patient had clinically negative lymph nodes. Histological examination showed malpighian cell proliferation arranged in clumps and coalescing lobules with focal areas of abrupt trichilemmal keratinization, with very marked atypies cyto-nuclear and an infiltrated fibrous stroma, suggesting proliferating trichilemmal tumor. Staging evaluation showed no metastase. The patient underwent wide surgical resection followed by in depth removal of extra tissue, without recurrence at 3-months’ follow-up

## Image en médecine

La tumeur trichilemmale proliférante, encore appelée kyste trichilemmal proliférant, est une tumeur annexielle maligne peu fréquente, qui se développe à partir des cellules de la gaine folliculaire externe ou le plus souvent à partir d'un kyste trichilemmal, après de multiples traumatismes et/ou d'inflammations itératives. Nous rapportons le cas d'une patiente âgée de 64 ans, sans antécédent, qui consultait pour une tumeur du cuir chevelu, évoluant progressivement depuis 18 mois. L'examen clinique montrait une masse tumorale ulcéro-bourgeonnante indolore, ferme, adhérente, de 12 cm de grand axe siégeant au niveau du vertex. Les aires ganglionnaires étaient libres. L'examen histologique montrait une prolifération cellulaire malpighienne agencée en massifs et en lobules coalescents centrés par une kératinisation abrupte selon un mode trichilemmal, avec des atypies cyto-nucléaires très marquées et un stroma infiltré fibreux, faisant évoquer une tumeur trichilemmale proliférante. Le bilan d'extension n'a pas objectivé de métastase. La patiente a bénéficié d'une exérèse chirurgicale large suivie d'une recoupe en profondeur, sans récidive avec un recul de 3 ans.

**Figure 1:**
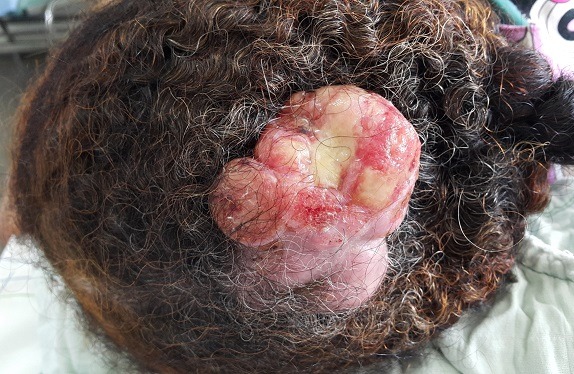
Masse tumorale exophytique, ulcéro-bourgeonnante, de 12 cm de grand axe, siégeant au niveau du vertex

